# TRIM19/PML Restricts HIV Infection in a Cell Type-Dependent Manner

**DOI:** 10.3390/v8010002

**Published:** 2015-12-23

**Authors:** Tanja Kahle, Bianca Volkmann, Kristin Eissmann, Alexandra Herrmann, Sven Schmitt, Sabine Wittmann, Laura Merkel, Nina Reuter, Thomas Stamminger, Thomas Gramberg

**Affiliations:** 1 Institute of Clinical and Molecular Virology, Friedrich-Alexander University Erlangen-Nürnberg, 91054 Erlangen, Germany; tanja_sanchez@yahoo.com (T.K.); bavolkma@viro.med.uni-erlangen.de (B.V.); kristin.eissmann@viro.med.uni-erlangen.de (K.E.); alexandra.herrmann@viro.med.uni-erlangen.de (A.H.); sven.schmitt3@gmx.de (S.S.); sabine.wittmann@viro.med.uni-erlangen.de (S.W.); lamerkel@viro.med.uni.erlangen.de (L.M.); nina.reuter@viro.med.uni-erlangen.de (N.R.); tsstammi@viro.med.uni-erlangen.de (T.S.); 2 Institute of Clinical Chemistry and Pharmacology, University Hospital Bonn, 53127 Bonn, Germany

**Keywords:** PML, TRIM19, ND10, HIV-1, retrovirus, intrinsic immunity

## Abstract

The promyelocytic leukemia protein (PML) is the main structural component of the nuclear matrix structures termed nuclear domain 10 (ND10) or PML nuclear bodies (PML-NBs). PML and ND10 structures have been shown to mediate an intrinsic immune response against a variety of different viruses. Their role during retroviral replication, however, is still controversially discussed. In this study, we analyzed the role of PML and the ND10 components Daxx and Sp100 during retroviral replication in different cell types. Using cell lines exhibiting a shRNA-mediated knockdown, we found that PML, but not Daxx or Sp100, inhibits HIV and other retroviruses in a cell type-dependent manner. The PML-mediated block to retroviral infection was active in primary human fibroblasts and murine embryonic fibroblasts but absent from T cells and myeloid cell lines. Quantitative PCR analysis of HIV cDNA in infected cells revealed that PML restricts infection at the level of reverse transcription. Our findings shed light on the controversial role of PML during retroviral infection and show that PML contributes to the intrinsic restriction of retroviral infections in a cell type-dependent manner.

## 1. Introduction

The promyelocytic leukemia protein (PML/TRIM19) is a member of the tripartite motif (TRIM) protein family and contains an amino-terminal TRIM motif consisting of a RING domain, two B-Boxes and a coiled coil domain [1,2]. Seven different PML isoforms have been described, all of which contain a TRIM motif but differ within their carboxyterminal sequences due to differential splicing. The PML isoforms I through VI are located in the nucleus of the cell, whereas isoform VII is exclusively found in the cytoplasm of the cells. Within the nucleus, PML is the major organizing component of nuclear domain 10 (ND10) structures. Within ND10, PML is modified by binding to the small ubiquitin-like modifier (SUMO), which leads to the stabilization of the ND10 structures and the recruitment of the permanently ND10-associated proteins Daxx and Sp100, as well as many other transiently associated proteins depending on exogenous influences and cellular conditions [3,4]. PML and ND10 structures have been implicated in a variety of different functions, including tumor suppression, DNA damage response, apoptosis, senescence, immune signaling, or antiviral restriction [5,6,7,8]. PML has been shown to negatively affect the replication of many DNA and RNA viruses [9]. Particularly well-studied is the role of PML as an intrinsic restriction factor during the replication of herpes viruses. The human herpes simplex virus type I (HSV-1), for example, encodes the immediate-early protein ICP0 to specifically degrade SUMOylated PML, underlining the importance of PML as antiviral restriction factor [10,11,12]. In the absence of ICP0, HSV-1 fails to undergo lytic replication due to the antiviral activity of ND10 structures.

The role of PML during retroviral infection, however, is less clear. PML belongs to the TRIM protein family, many members of which have been shown to affect the replication of retroviruses, like HIV-1 [13]. One of the best studied TRIM protein and a major host restriction factor is TRIM5α [14]. While human TRIM5α shows only little activity against HIV-1, TRIM5α from rhesus macaques efficiently inhibits HIV-1 infection. TRIM5α blocks HIV-1 by at least two distinct mechanisms early in the viral life cycle [15]. First, TRIM5α is believed to restrict infection by binding to incoming viral capsids, thereby inducing a premature and uncontrolled uncoating of the capsid [16]. In addition, a mechanistically ill-defined second block interferes with HIV replication prior to nuclear import of the viral cDNA. Furthermore, an indirect antiviral effect has been discussed, with TRIM5α serving as an intracellular pattern recognition receptor, which induces antiviral signaling upon binding to retroviral capsids [15,17]. Whether PML/TRIM19 is also active against HIV-1 infection, however, is controversially discussed. An initial study suggested that PML interferes with the early steps of HIV-1 replication in human cells and that HIV infection causes a re-localization of PML from PML-NBs into the cytoplasm shortly after infection [18]. Since arsenic trioxide (As_2_O_3_) promotes the degradation of PML, the authors used As_2_O_3_ to degrade PML and enhance HIV-1 infectivity. Although a second study by Berthoux *et al.* confirmed the enhancing effect of As_2_O_3_ on HIV infectivity, the authors also found that As_2_O_3_ enhanced infectivity independently of PML expression [19]. They could not detect an inhibitory effect of PML on HIV-1 infection in human or murine cells, nor observe a relocalization of PML upon infection [19,20]. Interestingly, recent work by Lusic *et al.* found that PML regulates HIV latency by colocalizing with integrated HIV provirus and that PML is able to induce the transcriptional silencing of the LTR promoter-driven gene expression during latency [21]. The reason for the discrepant results between the different studies is unclear at the moment but may be due to the different cell types used in the studies.

To shed light on the role of PML in HIV infection, we analyzed the antiviral activity of human and mouse PML on retroviral infectivity in various cell types. We found that the knockdown of PML, but not that of the PML-associated proteins Daxx and Sp100, enhances HIV infectivity in primary human fibroblasts (HFF). Although we could confirm the antiretroviral effect of PML in murine embryonic fibroblasts, we could not detect a significant antiviral effect of PML in human T cell lines or myeloid cell lines, indicating that the anti-HIV effect of PML is strongly cell type specific. Mechanistically, we found that the knockdown of PML is independent of viral accessory genes and the HIV LTR promoter and is also not restricted to HIV-1 but affects other retroviruses as well. In addition, we found that the knockdown of PML already enhances retroviral reverse transcription, indicating that the PML-mediated block to infection already occurs early during the viral life cycle.

## 2. Materials and Methods

### 2.1. Cells and Cell Culture

Primary human foreskin fibroblast (HFF) cultures were prepared from human foreskin tissue from multiple donors as described in previous studies [22] and were cultured in Dulbecco’s modified Eagle medium (DMEM) supplemented with 10% fetal bovine serum (FBS). PML knockout and wild-type murine embryonic fibroblasts (MEF) derived from C57BL6 mice were cultured in DMEM containing 10% FCS. 293T cells were kept in DMEM containing 10% FBS. The human T cell lines Jurkat, Molt4, CEM, and HuT78 as well as the myeloid cell lines U937 and THP-1 were cultured in RPMI 1640 medium containing 10% FBS. HFF cells stably overexpressing the PML isoforms I to VI from a retroviral vector and THP-1 cells stably expressing shRNAs directed against PML, Sp100, and Daxx were described in previous studies [23]. All cells transduced with lentiviral shRNA vectors were kept in medium containing additional 5 μg/mL puromycin.

### 2.2. Plasmids

Lentiviral vectors encoding short hairpin RNA targeting PML (pLVX-shPML), Daxx (pLVX-shDaxx), or SP100 (pLVX-shSP100) or scrambled shRNA (pLVX-shC) have been described in previous studies [23]. The env-deficient HIV-1 reporter plasmid pNL43-E^−^-GFP encodes the EGFP reporter gene in place of the nef open reading frame and has been described in previous studies [24]. The reporter plasmid pNL43-E^−^-CMVGFP expresses GFP under control of an additional CMV promoter within nef [24]. The HIV-1 reporter construct pNL4-3eGFP containing a Matrix-eGFP fusion protein has been described in previous studies and was donated by B. Schmidt [25]. The lentiviral transfer vector pWPI was donated by Didier Trono (Addgene plasmid # 12254) and expresses the EGFP reporter gene under the control of the cellular promoter EF1α. The env-deficient retroviral reporter constructs pNL-luc3-E^−^ (HIV-luc) [26], pSIVmac239-luc-E^−^ (SIV-luc) [24], pSARM-EGFP (MPMV-GFP) [27], and pMXSfi-EGFP (MLV-GFP) [24] have been described in previous studies. The lentiviral packaging vector pΔR8.91 expresses HIV gagpol and has also been described in previous studies [28].

### 2.3. Virus Preparation

ShRNA-containing particles were produced by cotransfection of 293T cells with the respective pLVX-shRNA vector, the lentiviral packaging plasmid pΔR8.91, and the pVSV-G vesicular stomatitis virus glycoprotein expression plasmid at a mass ratio of 2:2:1 using calcium phosphate coprecipitation. Reporter viruses were produced in 293T cells cotransfected with env-deficient reporter virus plasmids and pVSV-G. HIV-GFP and HIV-CMVGFP were generated by cotransfecting pNL43-E^−^-GFP or pNL43-E^−^-CMVGFP together with pVSV-G at a mass ration of 4:1. Fluorescently labeled HIV-MAGFP particles were produced by cotransfecting pNL-43eGFP and VSV-G at a mass ratio of 4:1. HIV-GFP Δacc was generated by cotransfection of pWPI, pΔR8.91 and pVSV-G at a mass ration of 2:2:1. HIV-1-Luciferase (HIV-luc) reporter virus was generated by cotransfection of pNL.Luc3-E^−^ and pVSV-G at a mass ratio of 5:1. MLV-GFP reporter virus was produced by transfection with pMX-GFP, the packaging plasmid pHIT-60, and pVSV-G at a mass ratio of 4:2:1. MPMV-GFP reporter virus was generated by transfection of pSARM-EGFP and pVSV-G at a mass ratio of 4:1. After 6 h, the culture medium of the transfected cells was replaced. Supernatants were harvested 48 h posttransfection, passed through 0.4-μm-pore size filters, aliquotted and frozen at −80 °C.

SIV and HIV luciferase reporter viruses were normalized upon infection of 293T cells by luciferase assay according to the manufacturer’s instructions (Promega, Fitchburg, WI, USA). Therefore, 1 × 10^4^ 293T cells were infected with 10, 30, and 50 μL of luciferase reporter virus. After 72 h, infected cells were lysed, and luciferase activity was quantified as counts per second (cps). Similarly, GFP reporter viruses were titered on 293T cells and analyzed 72 h postinfection via flow cytometry. Lentiviral shRNA vectors were normalized on p24 capsid protein content determined in ELISA assays, which were conducted by the diagnostic section of the institute.

### 2.4. Reporter Virus Infection Assays

For HFF and MEF infections, 1 × 10^5^ cells were seeded into 6-well plates and transduced with GFP reporter viruses the next day at a MOI of 0.3 if not indicated otherwise. To assess the role of sodium arsenite during HIV infection, HFF cells were treated with the indicated amounts (1 to 4 μM) of NaAsO_2_ (Sigma, St. Louis, MO, USA) for 12 h prior to infection and 24 h postinfection. For luciferase reporter virus infections, 2 × 10^4^ HFF or MEF cells were infected with increasing amounts of luciferase reporter virus (cps) unless otherwise indicated. Human T cell lines and myeloid cells were infected with GFP reporter viruses at the indicated MOI or with increasing amounts of luciferase reporter virus. After six hours, medium of the transduced cells was replaced. The number of infected cells was determined after 72 h by flow cytometry, and luciferase activity in infected cells (counts per second, cps) was quantified via luciferase assay 72 h postinfection.

### 2.5. Quantitative Real-Time PCR (qPCR)

HFFs or MEFs were infected at a MOI of 1, with virus stocks treated for 1 h with 50 U of Benzonase/mL (Sigma) to remove contaminating plasmid DNA. At 14 h prior to infection, 25 μM reverse transcriptase inhibitor (AZT) was added to one well to control for residual plasmid DNA contamination. At 12 h, 24 h, and 48 h postinfection, total cellular DNA was isolated using a QIAamp Blood DNA Kit (Qiagen, Chatsworth, CA, USA). Reverse transcripts in 125 ng (HFF) or 250 ng (MEF) of total DNA were quantitated by qPCR with an ABI Prism 7300 (Applied Biosystems). The primer pairs used to detect HIV late reverse transcription products were MH531 (5'-TGTGTGCCCGTCTGTTGTGT), MH532 (5'-GAGTCCTGCGTCGAGAGA) and the TaqMan probe LRT-P 5'-(FAM)-CAGTGGCGCCCGAACAGGGA-(TAMRA)-3'. To detect HIV 2LTR circles, MH535 (5'-AACTAGGGAACCCACTGCTTAAG) and MH536 (5'-TCCACAGATCAAGGATATCTTGTC) together with the TaqMan probe MH603 (5'-(FAM)-ACACTACTTGAAGCACTCAAGGCAAGC TTT-(TAMRA)-3') were used. Standard curves were generated using serially diluted proviral and 2-LTR plasmids.

### 2.6. Vpr-BLaM Entry Assay

β-Lactamase-containing virions were produced by cotransfection of 293T cells with the proviral plasmid pNL4-3luc E^−^, pMM310, a vector that encodes a β-lactamase-Vpr fusion protein, and pVSV-G at a mass ration of 2:2:1. Virus stocks were normalized on equal infectivity and used to transduce HFF shC or shPML cells at a MOI of 1. After 5 h, the cells were washed with PBS and incubated with 2 mM of the BLaM substrate CCF2 (Gene Blazer Kit, Aurora Biosciences, San Diego, CA, USA), 1% probenecid in 1.0 mL serum-free DMEM containing 10 mM Hepes at 25 °C. After 18 h, cells were washed, fixed in 1% paraformaldehyde for 10 min, and analyzed by flow cytometry. Cells containing cleaved substrate were detected with a 424/44-nm band pass filter (blue) and uncleaved substrate was detected with a 516/20-nm band pass filter (green) by flow cytometry.

### 2.7. Immunoblot Analysis

All cells were lysed in NP40 lysis buffer (10 mM TrisHCl (pH 7.5), 150 mM NaCl, 2 mM EDTA, 0.5% NP-40, Halt Protease Inhibitor). Lysates were quantified by Bradford assay (Carl Roth, Karlsruhe, Germany) and 30 μg per sample were separated by SDS-PAGE, transferred to PDVF membranes and probed with different primary antibodies. To detect PML in immunoblots, the rabbit polyclonal antibodies A301-167A and A301-168A (Bethyl-Laboratories, Montgomery, TX, USA) were used in combination. To detect human Daxx, the rabbit monoclonal antibody E94 (BioCat, Heidelberg, Germany) was used, and, to detect SP100, the rabbit polyclonal AB11377-1-AP (Acris, San Diego, CA, USA) was used. As loading control, membranes were probed with murine antibodies directed against the house keeping genes GAPDH, HSP90, or β-Actin. The membranes were then probed with anti-mouse or anti-rabbit horseradish peroxidase (HRP)-labeled secondary antibody (goat, GE Healthcare) and visualized using HRP substrate (Pierce, Rockford, IL, USA).

### 2.8. Immunofluorescence Analysis

For indirect immunofluorescence analysis, adherent cells were grown on poly-lysine-coated coverslips in 24-well dishes (2.5 × 10^4^ cells/well). After 24 h, cells were fixed with 4% paraformaldehyde for 20 min at room temperature. For the analysis of PML localization upon HIV infection, 3.0 × 10^5^ HFF cells were grown on coverslips for 24 h in 6 well dishes, infected with HIV-MAGFP at a MOI of 3, and harvested at the indicated time points. Non-adherent cells were seeded at 1 × 10^5^ cells/50 μL in single wells of well-containing cover slips, dried, and fixed in 50 μL 4% paraformaldehyde (Applichem, Darmstadt, Germany) for 60 min. Subsequently, all cells were washed with PBS three times followed by permeabilization using a 0.4% saponine (Applichem) solution on ice for 20 min. After another washing step, cells were stained with primary antibodies against PML, SP100, or Daxx overnight at 4 °C. The corresponding secondary antibody dilutions were used to incubate the cells for 60 min at room temperature. Finally, cells were stained with DAPI nucleic acid stain (4′,6-diamidino-2-phenylindole, Invitrogen, Carlsbad, CA, USA) and mounted using Fluoromount mounting medium (Sigma). Samples were analyzed using the Leica TCS SP5 confocal laser-scanning microscope (Leica, Wetzlar, Germany), and the resulting images were processed using Photoshop software (Adobe, San José, CA, USA).

## 3. Results

**Figure 1:**
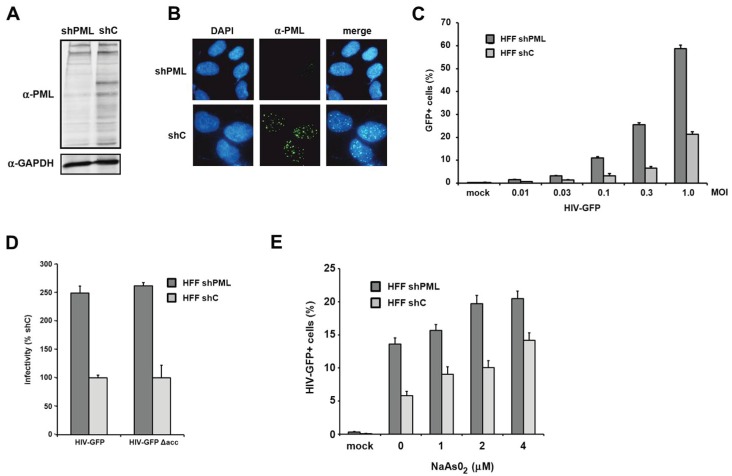
The knockdown of PML correlates with enhanced HIV-1 infectivity in HFF. (**A**) Lysates from HFF cells stably expressing shRNA directed against PML (shPML) or control shRNA (shC) were analyzed on an immunoblot probed with anti-PML antibodies or anti-GAPDH MAb; (**B**) Cellular localization of PML in HFF shC and shPML cells. HFFs were seeded onto coverslips, washed, and probed with antibodies against PML; (**C**) HFF shPML and shC cells were infected in triplicates with increasing MOI (multiplicity of infection) of VSV-G pseudotyped HIV-GFP reporter viruses or medium (mock); (**D**) HFF shPML and shC cells were infected with HIV-GFP reporter virus coding for all viral accessory proteins but nef (HIV-GFP) or with virus lacking all accessory protein (HIVGFP Δacc); (**E**) HFF shPML and shC cells were treated with increasing concentrations of sodium arsenite (NaAsO_2_) for 36 h and infected with HIV-GFP reporter virus at a MOI of 0.3. HIV infectivity was determined 72 h postinfection by flow cytometry. The data are presented as the average of triplicates with error bars indicating the standard deviation. The results shown are representative of three independent experiments. Mock: not infected.

PML knockdown correlates with enhanced retroviral infectivity in primary human fibroblasts. To clarify the role of PML/TRIM19 during retroviral replication, we infected primary human foreskin fibroblasts (HFF) with retroviral reporter viruses. We used HFF cells since they are a well-characterized model system to analyze the antiviral effect of PML on the replication of different viruses, e.g., herpesviruses [29]. To analyze the effect of PML on retroviral replication, we generated knockdown cell lines by transducing HFF cells with a lentiviral vector expressing shRNA targeting PML (shPML) or control shRNA (shC). We found that the knockdown of PML efficiently reduced the amount of total PML protein in shPML cells when analyzed by immunoblotting (Figure 1A). The various bands visible in the lysate of control cells indicate the different PML isoforms as well as differentially modified proteins. We found that the expression of shRNA against PML efficiently down-regulates most PML variants (Figure 1A). In HFF shC cells, we found PML to be localized in nuclear speckles, the previously described ND10 structures (Figure 1B). To analyze the effect of the PML knockdown on HIV infectivity, we transduced shC- or shPML-expressing HFFs with increasing amounts of VSV-G pseudotyped HIV-GFP reporter virus and quantified the number of GFP expressing cells three days postinfection by flow cytometry (Figure 1C). We found a three- to four-fold enhancement of HIV infectivity in HFFs lacking PML compared to shC cells. This suggests that the expression of PML restricts HIV infection during the early phases of the viral life cycle in primary human fibroblasts. The inhibitory effect of PML on HIV infectivity did not change over a wide range of multiplicity of infections (MOIs), suggesting that the antiviral effect of PML is not saturable by high viral loads (Figure 1C). Many lentiviral accessory proteins play an important role in overcoming intrinsic antiviral restrictions. Vpu, for example, is counteracting the Tetherin-mediated block, Vif is blocking the APOBEC3-mediated restrictions, and Vpx/Vpr disarms the SAMHD1-mediated inhibition of retroviral infection. We therefore wondered whether the accessory proteins Vpu, Vif, or Vpr of HIV-1 would interfere with the PML-mediated reduction of infectivity (Figure 1D). Thus, we compared the infectivity of HIV-GFP reporter viruses encoding all accessory proteins but nef (HIV-GFP) or lacking all accessory proteins (HIV-GFP Δacc) on HFF shC and shPML cells. Three days postinfection, we found that both viruses infect HFF cells almost to the same levels. Compared to shPML cells, we found that the infectivity of HIV-GFP was strongly reduced in shC cells independently of HIV accessory proteins. It is therefore unlikely that HIV-1 encodes an accessory protein that counteracts the PML-mediated restriction (Figure 1D). Turelli *et al.* have shown that the degradation of ND10 structures upon arsenic treatment enhanced HIV-1 infectivity [18]. To determine whether treatment with arsenical compounds mimics the shRNA-mediated knockdown of PML in HFF cells, we treated shPML and shC HFF with increasing amounts of sodium arsenite (NaAsO_2_), which has been shown to down-regulate PML protein level even more efficiently than As_2_O_3_ [30]. Sodium arsenite was added 12 h prior to infection with the HIV-GFP reporter virus at a MOI of 0.3 (Figure 1E). After additional 24 h, the NaAsO_2_-containing medium was replaced to minimize cytotoxic effects, and viral infectivity was determined 72 h postinfection by flow cytometry. We were able to confirm the findings of Turelli *et al.* and found that arsenical compound treatment increases HIV infectivity on shC cells. On shC cells, incubation with 4 μM arsenite enhanced viral infection by 2.4-fold and up to the level of viral infectivity on shPML-expressing HFFs, suggesting that the shRNA-mediated knockdown of PML can indeed be mimicked by sodium arsenite treatment. However, we also observed a slight enhancement of HIV infection upon treatment of shPML cells (up to 1.5-fold). The enhancement in shPML cells might be due to the inactivation of residual PML molecules and ND10 structures or by pleiotropic effects of arsenical compounds in the target cells, making the shRNA-mediated knockdown of PML the preferred tool to analyze the role of PML during retroviral replication.

**Figure 2:**
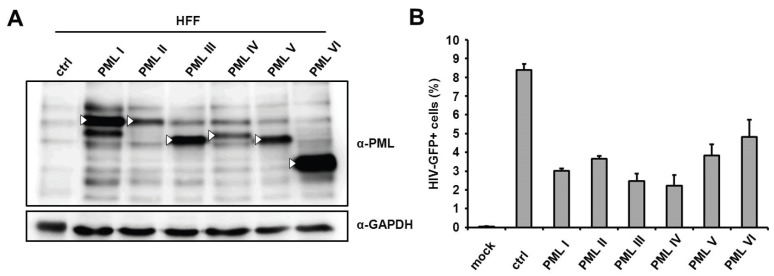
The separate overexpression of different PML isoforms reduces HIV-1 infectivity in HFF. (**A**) Lysates from HFF cells stably overexpressing the PML isoforms I to VI or control cells (ctrl) were analyzed by immunoblot probed with anti-PML antibodies or anti-GAPDH MAb. White triangles mark the predicted size of the PML isoforms; (**B**) PML isoform I to VI expressing HFF cells or control cells (ctrl) were either not infected (mock) or transduced with VSV-G pseudotyped HIV-GFP reporter viruses at a MOI of 0.3. HIV infectivity was determined 72 h postinfection by flow cytometry. The data are presented as the average of triplicates, with error bars indicating the standard deviation. The results shown are representative of four independent experiments. Mock: not infected.

Since we found that the knockdown of PML enhanced HIV-GFP infection in HFFs, we next asked whether the overexpression of PML would be able to reduce infectivity in these cells. We therefore employed previously described HFF cells, which stably and separately overexpress six of the seven known PML isoforms (I to VI) from a retroviral vector (Figure 2A) [23]. Upon infection with HIV reporter virus, we found that the overexpression of the different isoforms reduced infectivity by approximately 1.5- to 3-fold in HFFs (Figure 2B), suggesting that the overexpression of PML also has a moderate inhibitory effect on HIV infections. However, also in part due to the heterogenic expression levels of the isoforms, we could not associate the antiviral effect of PML with a specific isoform.

**Figure 3:**
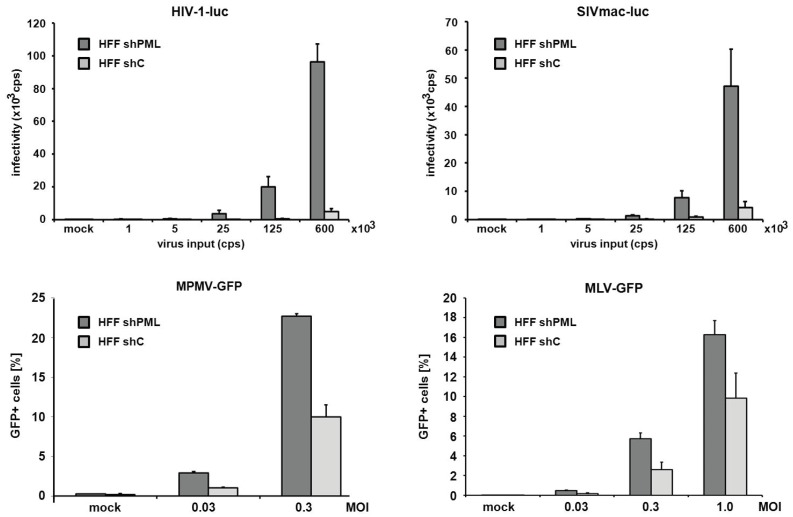
PML blocks the infection of diverse retroviruses. HFF shPML and shC cells were infected in triplicates with increasing amounts of VSV-G pseudotyped reporter viruses or medium (mock). Cells were inoculated with increasing amounts (counts per second; cps) of HIV luciferase-encoding (HIV-luc) or SIV luciferase encoding (SIVluc) reporter virus. Infectivity was determined 72 h postinfection by a luciferase assay and is depicted as counts per second (cps). For MPMV-GFP and MLV-GFP infections, HFF shPML and shC cells were infected with increasing MOI (multiplicity of infection) of VSV-G pseudotyped GFP reporter viruses. Infectivity was determined 72 h postinfection by flow cytometry. Error bars indicate the standard deviation of triplicate infections. The results shown are representative of three independent experiments.

Next, we asked whether the inhibitory effect of PML is specific for HIV-1 or whether other retroviruses are restricted by PML as well. We therefore transduced HFF shC and shPML cells with increasing amounts of different single-round reporter viruses. Infection with a simian immunodeficiency virus (SIV) expressing a luciferase reporter gene, a murine leukemia reporter virus (MLV) expressing GFP, a γ-retrovirus, a GFP reporter virus based on Mason-Pfizer monkey virus, a δ-retrovirus, showed similar results to the HIV-luciferase infection (Figure 3). We found that all reporter viruses tested infected shPML cells about 2- to 4-fold more efficiently than HFF control cells, demonstrating that the antiviral effect of PML is not specific to HIV-1 but more generalized and affects a broader range of retroviruses.

**Figure 4:**
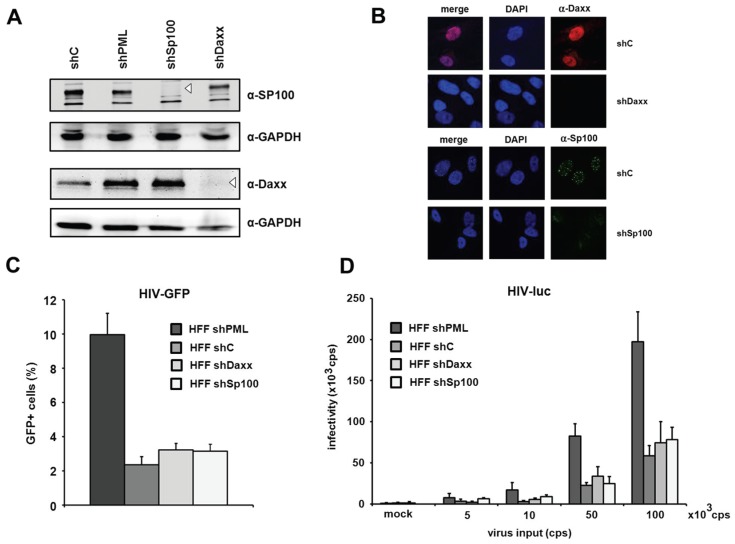
The knockdown of PML but not of Daxx or Sp100 affects HIV infectivity. (**A**) Lysates from HFFs expressing shRNA directed against, Daxx (shDaxx), Sp100 (shSp100), or control shRNA (shC) were analyzed via immunoblot. Membranes were probed with antibodies directed against Daxx, Sp100 or the loading control GAPDH; (**B**) Cellular localization of Daxx and Sp100 in HFF cells. Cells were seeded onto coverslips, washed and probed with antibodies against Daxx or Sp100; (**C**) HFF shPML, shDaxx, shSp100, and shC cells were infected in triplicates with VSV-G pseudotyped HIV-GFP at a MOI of 1 or medium (mock). Infectivity was determined 72 h postinfection by flow cytometry; (**D**) HFF shPML, shDaxx, shSp100, and shC cells were infected in quadruplicates with increasing amounts of HIV-luc (cps). Infectivity was determined 72 h postinfection by a luciferase assay and is depicted as 10^3^ cps. One of three independent experiments is shown. Error bars indicate the standard deviation of triplicate infections.

The knockdown of Daxx and Sp100 does not affect HIV infectivity in HFFs. PML is the main structural component of ND10 structures. However, aside from PML, the constitutively ND10-associated proteins Sp100 and Daxx have also been shown to be active against a variety of different viruses. Since the knockdown of PML also abolishes ND10 formation and thereby changes the localization of Sp100 and Daxx, it is conceivable that this change might also affect a potential antiviral activity of Sp100 or Daxx. Therefore, we decided to determine whether the ND10-associated proteins Sp100 and Daxx also affect HIV-1 infectivity and generated HFF cells, in which Daxx or Sp100 protein expression is suppressed by shRNA (Figure 4A). In immunofluorescence analysis, we found Sp100 to be localized within ND10s in the nucleus of shC HFFs, while the speckled staining was missing in shSp100 cells (Figure 4B). We also found the nuclear staining of Daxx to be absent from shDaxx cells. In shC HFFs, the localization of Daxx was not confined to ND10 structures but evenly distributed within the nucleus, suggesting that Daxx localizes not exclusively to ND10 structures (Figure 4B). Upon infection of knockdown and control shRNA expressing HFF cells, we detected an increase in HIV reporter virus infectivity in shPML HFFs, but not in shDaxx or shSp100 cells when compared to the infectivity in control cells (Figure 4C). Similarly, the infection with increasing amounts of the HIV-luciferase virus was enhanced only in the absence of PML but not in the absence of Sp100 or Daxx (Figure 4D). Thus, HIV infectivity in HFFs does not seem to be affected by the ND10-associated proteins Sp100 or Daxx but only by the main structural ND10-component PML.

**Figure 5:**
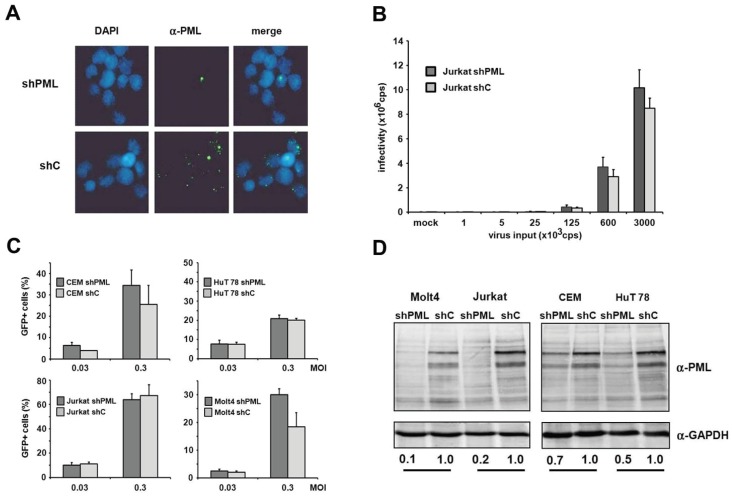
PML does not affect HIV-1 infectivity in T cells. (**A**) Jurkat shC or shPML cells were probed for immunofluorescence analysis using an anti-PML antibody mix and DAPI for nuclear staining; (**B**) Jurkat shC and shPML cells were infected with increasing amounts of VSV-G/HIV-luc reporter virus or medium (mock). Luciferase activity was determined 72 h postinfection and is depicted as counts per second (cps); (**C**) Jurkat, Molt4, CEM, and HuT78 T cell lines harboring shRNA targeting PML or control shRNA were infected with HIV-GFP at the MOIs 0.03 and 0.3. HIV-GFP infectivity was quantified after 72 h by flow cytometry. Error bars indicate the standard deviation of triplicate infections; (**D**) Cell lysates of the different shC and shPML T cell lines were analyzed by immunoblot and probed with anti-PML and anti-GAPDH antibodies. Numbers indicate the PML expression levels in shPML cells relative to shC cells. PML protein levels were quantified using the AIDA imaging software (Raytest) and were normalized to GAPDH expression.

HIV infectivity is not enhanced in the absence of PML in T cells and myeloid cells. Human T cells and macrophages are *bona fide* target cells of HIV-1 infection *in vivo*. Unfortunately, we found the manipulation of endogenous PML levels by small interfering RNA in primary cells to be rather difficult and very inefficient. Thus, to determine whether PML affects infection in HIV target cell types, we analyzed HIV infectivity in T cell lines and myeloid cells in the absence or presence of PML. First, we generated PML knockdown (shPML) and control cells (shC) based on four different T cell lines, CEM, HuT78, Jurkat and MOLT4, which stably express shRNA directed against PML or control shRNA from a lentiviral vector. In Jurkat T cells, we found that overexpression of shRNA targeting PML efficiently reduced the number of nuclear ND10 structures compared to shC cells, indicating an efficient reduction of PML protein level in Jurkat shPML cells (Figure 5A). Next, we transduced shC and shPML Jurkat T cells with increasing amounts of HIV-luciferase reporter virus and analyzed viral infectivity 72 h postinfection by luciferase activity in the target cells. However, in contrast to HFFs, we could not detect a significantly enhanced HIV reporter virus infection in Jurkat cells upon PML knockdown (Figure 5B). To exclude any cell type-specific limitations regarding the antiviral effect of PML in Jurkat cells, we generated three additional T cell lines stably expressing shPML or control shRNA (Figure 5C,D). The efficacy of the PML knockdown varied between the different cell lines. While in CEM and Hut78 T cells the knockdown of PML was only moderately effective, the PML protein level in Jurkat and Molt4 were reduced by 80% and 90%, respectively. However, upon infection we could not detect a significant difference in HIV infectivity between shC and shPML cells for any of the tested T cell lines. Together, our results suggest that PML does not affect HIV-1 infectivity in T cells—at least not in the T cell lines tested.

**Figure 6:**
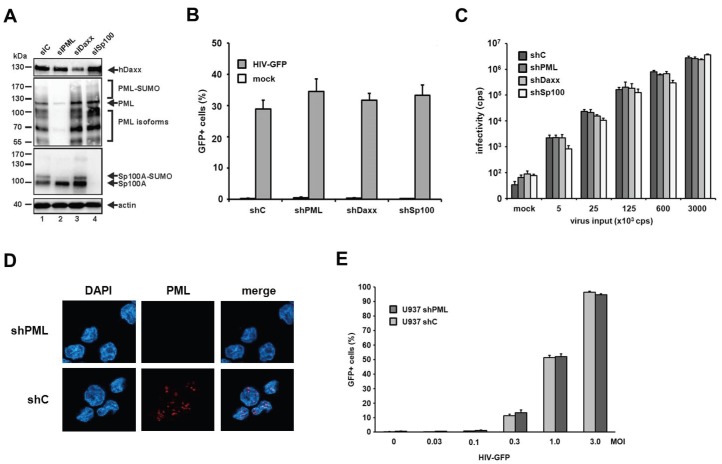
The knockdown of PML does not enhance HIV-1 infectivity in myeloid cells. (**A**) THP-1 shC, shPML, shDaxx, or shSp100 cells lysates were separated by SDS-PAGE, blotted, and probed with antibodies direct against PML, Daxx, Sp100, or the loading control Actin; (**B**) THP-1 shC, shPML, shDaxx, or shSp100 cells were infected with HIV-GFP at an MOI of 0.3 or (**C**) with increasing amounts of HIV-luciferase reporter virus. Luciferase or GFP expressing cells were quantified 72 h postinfection by a luciferase assay (counts per second, cps) or flow cytometry (percent GFP-positive cells); (**D**) U937 shC or shPML cells were probed for immunofluorescence analysis using an anti-PML antibody mix and DAPI for nuclear staining; (**E**) U937 shC or shPML cells were infected with increasing MOIs of HIV-GFP. Infectivity was quantified 72 h postinfection by flow cytometry. Each result is representative of at least three independent experiments. Error bars indicate the standard deviation of triplicate infections.

Next, we generated myeloid THP-1 cell lines expressing shRNAs targeting PML, Sp100, Daxx, or control shRNA [23]. We confirmed the efficiency of the knockdown by immunoblot analysis using the respective antibodies (Figure 6A). We employed the THP-1 cells expressing the different shRNAs in HIV-GFP and HIV-luciferase reporter virus infection assays (Figure 6B,C). In contrast to HFF cells, HIV-GFP infection of THP-1 cells did not reveal any differences in susceptibility between shPML cells and THP-1 control cells (Figure 6B). In addition, the shRNA-mediated knockdown of Sp100 and Daxx in THP-1 cells did not affect HIV-GFP infection of these cells (Figure 6B). To test a second myeloid cell line, we infected U937 shC and shPML cells with increasing amounts of HIV-GFP reporter virus (Figure 6D,E). However, similar to THP-1 cells, we could not detect an enhancement of HIV infection upon knockdown of PML in comparison to control cells. Thus, our results indicate that PML does not play a role during early HIV infection events in myeloid cells.

**Figure 7:**
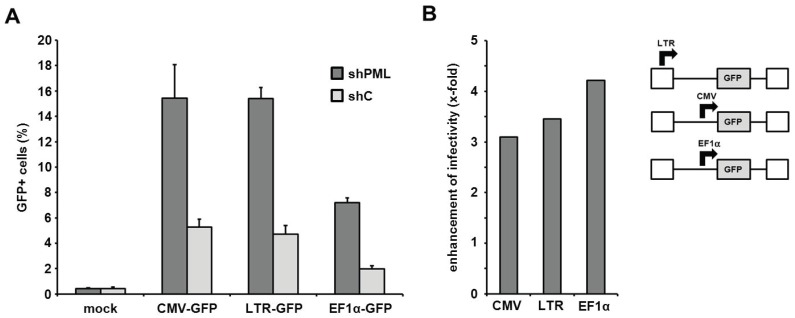
The PML-mediated block to HIV infection acts independently of the LTR promoter. (**A**) HFF shPML and shC cells were transduced with the indicated HIV-GFP virus expressing the reporter gene under the control of the HIV LTR promoter (LTR-GFP), the herpesviral CMV promoter (CMV-GFP), or the cellular EF1α promoter (EF1α-GFP) at a MOI of 0.3. Infectivity was determined 72 h postinfection by flow cytometry. Error bars indicate the standard deviation of triplicate infections. (**B**) Same as in (**A**) but blotted as enhancement of HIV infectivity in shPML cells over shC cells.

The PML-mediated block to infection is independent of the HIV promoter activity. Next, we aimed at determining the step of the retroviral life cycle that is affected by PML. Recently, PML has been show to colocalize with integrated HIV proviruses in latently infected cells and to down-regulate transcription from the proviruses, thereby contributing to the transcriptional silencing during HIV latency [21]. We therefore asked whether the LTR-mediated transcription of the virus-encoded reporter genes is affected by the presence of PML and whether this influences the outcome of our infection assays. Thus, we transduced HFF shC and shPML cells with three different HIV reporter viruses, which each express the GFP reporter gene under control of different promoters (Figure 7). The different reporter viruses express GFP under control of the HIV LTR promoter (LTR-GFP), the major immediate-early enhancer/promoter from human cytomegalovirus (CMV-GFP), or the cellular human elongation factor 1 alpha promoter (EF1α-GFP). Upon infection, we found that the knockdown of PML (shPML) enhanced the infectivity of all three reporter viruses independently of the promoter when compared to control HFFs (shC) (Figure 7A). The absence of PML enhanced the infectivity of all three viruses by 3- to 4-fold, suggesting no promoter specific differences in PML restriction (Figure 7B). Although we cannot exclude that overall cellular transcription levels are negatively influenced by the presence of PML, our analysis indicates that the inhibitory effect of PML on retrovirus infection is not specific to the HIV LTR promoter activity.

**Figure 8:**
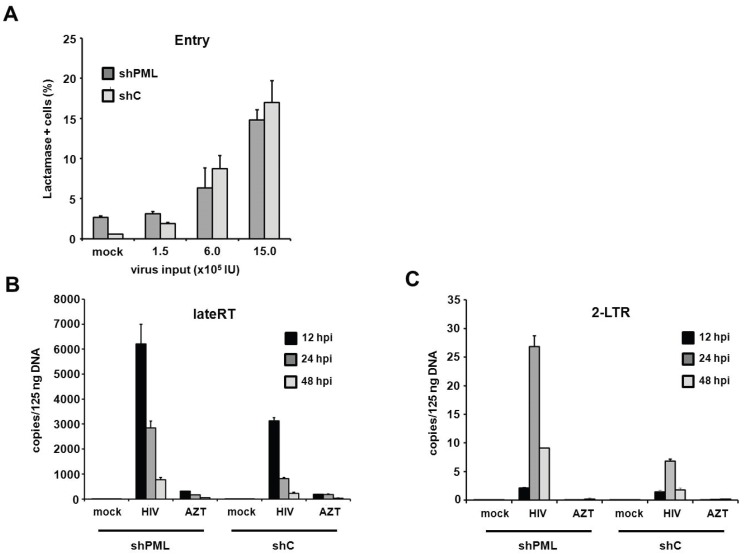
PML restricts HIV at the level of reverse transcription in HFF cells. (**A**) HFF shC or shPML cells were infected with increasing amounts of NL4-3 BlaM-Vpr. After 5 h, virus was removed, and the cells were loaded with the BlaM substrate CC2F for 18 h at 25 °C. Subsequently, cells were washed, fixed and analyzed by flow cytometry. The frequency of substrate cleavage following virus entry is depicted as percent lactamase positive cells; (**B**) HFF shC or shPML cells were infected with control supernatant (mock) or with HIV-GFP reporter virus. Total cellular DNA of the infected cells was isolated 12 h, 24 h, and 48 h postinfection (hpi) and used as a template in qPCR to amplify late reverse transcriptase products or (**C**) 2-LTR circles. AZT was added to one well prior to infection to control for contaminating proviral plasmid. The data are presented as the average of triplicates with error bars indicating the standard deviation. One out of three independent experiments is shown.

PML restricts HIV infection at the level of reverse transcription in human fibroblasts. Since the antiviral effect of PML acts upstream of HIV transcription, we next wanted to make sure that membrane fusion between the reporter viruses and the target cells is not altered by the down-modulation of PML. We therefore infected HFF cells with HIV reporter viruses that specifically incorporated a Vpr-β-Lactamase fusion protein (Vpr-BlaM) [31]. Upon successful membrane fusion, Vpr-BlaM gets released into the cytoplasm and cleaves the fluorescent substrate used to probe the target cells (CCF2). The resulting shift in the wavelength emitted from the cleaved substrate was analyzed 5 h postinfection by flow cytometry (Figure 8A). Upon transduction with increasing amounts of Vpr-BlaM loaded HIV reporter viruses, we could not detect a significant difference in particle uptake between shC and shPML cells, indicating that the knockdown of PML does not affect the entry process of the reporter viruses. To further characterize the impact of PML on HIV infectivity, we sought to assess the effect of PML on reverse transcription and nuclear import. We therefore infected HFF shC and shPML cells with HIV reporter virus and determined the number of reverse transcribed viral DNA molecules present at various time points postinfection (Figure 8B,C). We isolated total cellular DNA of infected HFFs 12 h, 24 h, and 48 h postinfection and determined the accumulation of viral DNA by quantitative PCR (qPCR) using primer pairs specific to late reverse transcription (late RT) and 2-LTR circles, as a surrogate marker for nuclear import. In both cell types, we found that late RT products peaked around 12 h and diminished at 24 and 48 h postinfection. As expected, copy numbers of the nuclear import marker 2-LTR circles peaked later, at 24 h postinfection, and were again reduced at 48 h postinfection. Interestingly, we found 2- to 3-fold elevated levels of late RT products in shPML cells at all three time points in comparison to shC cells (Figure 8B). This finding indicates that the knockdown of PML already relieves a block early during the viral life cycle, at or prior to reverse transcription. Fittingly, we also found a 3- to 4-fold increase in 2-LTR circles in shPML cells compared to control cells, suggesting elevated numbers of viral DNA molecules in the nucleus of shPML cells compared to control cells. An AZT control was included in the infections to control for contaminating input plasmid DNA. Together, these results suggest that PML already inhibits viral infectivity at or during reverse transcription and the subsequent nuclear import of viral cDNA. Although we cannot completely exclude additional, later mechanisms, the PML-mediated block to RT and nuclear import mirrors the magnitude of restriction measured in HIV-GFP infectivity assays, suggesting that this early restriction is the most relevant block in HIV reporter virus infection.

**Figure 9:**
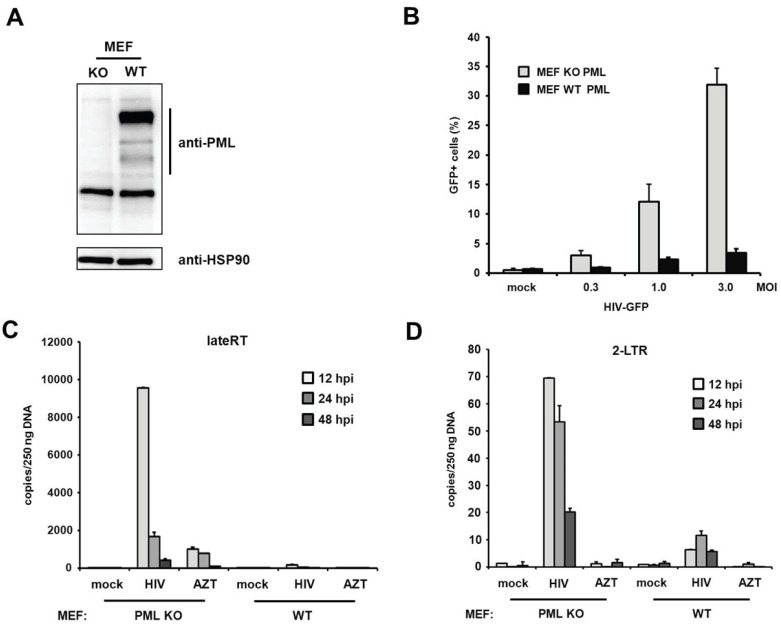
PML restricts HIV reverse transcription in MEFs. (**A**) Lysates from wild-type (WT) or PML knockout (KO) MEFs were immunoblotted and probed with anti-PML polyclonal antibodies or anti-HSP90 MAb; (**B**) WT or KO MEFs were infected with increasing MOI of VSV-G/HIV-GFP reporter virus. Infectivity was determined 72 h postinfection by flow cytometry; (**C**) WT or KO MEFs were infected with control supernatant (mock) or with HIV-GFP reporter virus at a MOI of 1. Total cellular DNA of the infected cells was isolated 12 h, 24 h, and 48 h postinfection (hpi) and used as template in qPCR to amplify late reverse transcriptase products or (**D**) 2-LTR circles. AZT was added to one well prior to infection to control for contaminating proviral plasmid. The data are presented as the average of triplicates with error bars indicating the standard deviation. One out of three independent experiments is shown.

PML blocks viral infectivity in murine embryonic fibroblasts. Within this study we found that the knockdown of PML enhances HIV-1 infectivity in primary human fibroblasts. Interestingly, a previous study using murine fibroblasts (MEFs) could not detect any differences in HIV infectivity between MEFs from PML KO and WT mice [19]. To determine whether PML restricts retroviral infection in murine fibroblasts as well, we transduced MEFs from PML knockout (KO) and wild-type (WT) mice with increasing amounts of VSV-G-pseudotyped HIV reporter virus expressing GFP under the control of a CMV promoter (Figure 9A,B). In contrast to the previous study, we found that the HIV infectivity in MEFs is enhanced by up to 10-fold in the absence of PML, which is an even stronger PML phenotype than in human fibroblasts. To confirm our finding that PML blocks retroviral infectivity in MEFs, we asked whether murine PML also inhibits HIV reverse transcription, similar to PML in human fibroblasts. We therefore transduced MEFs from KO or WT mice with HIV reporter virus and determined the number of viral RT products and 2-LTR circles at various time points postinfection (Figure 9C,D). Similar to the situation in human fibroblasts, we found that the knockout of PML already enhanced the number of late RT products as well as 2-LTR circles upon infection of MEFs. We found that in MEFs late RT products also peak around 12 h postinfection and are about 10-fold more abundant in PML KO cells compared to WT cells (Figure 9C). Also, the number of 2-LTR circles was enhanced in these cells by approximately 10-fold compared to WT cells (Figure 9D). As for HFFs, we found that the absence of PML in murine fibroblasts also releases an early block to HIV infection that otherwise acts during or prior to reverse transcription of the viral genome.

**Figure 10:**
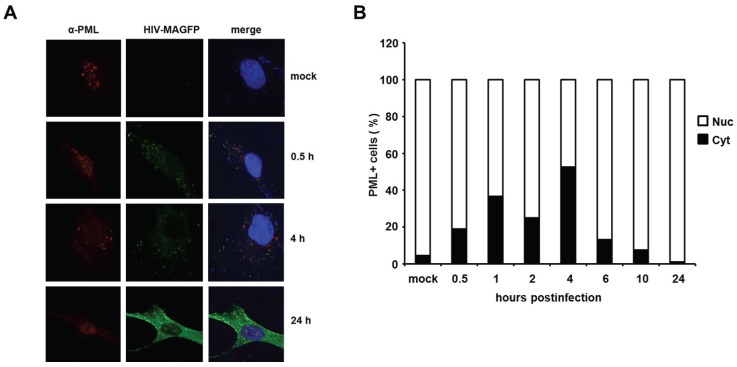
HIV infection induces a temporary relocalization of ND10 structures to the cytoplasm. (**A**) WT HFF cells were seeded on coverslips and infected with Matrix-GFP-containing HIV reporter virus at a MOI of 3 (HIV-MAGFP), or left untreated (mock). At 0.5 h, 1 h, 2 h, 4 h, 6 h, 10 h, and 24 h postinfection, the cells were extensively washed and probed for immunofluorescence analysis using anti-PML antibodies and DAPI for nuclear staining. The presence of HIV-MAGFP particles (green), the PML localization (red), and DAPI staining (blue) was analyzed at the different time points postinfection by confocal microscopy. Exemplary analysis of uninfected cells (mock) and HIV-GFP infected cells at 0.5 h, 4 h, and 24 h postinfection are depicted; (**B**) Statistical analysis of the PML localization in HIV-GFP-positive cells. For each indicated time point, the cellular localization of PML/ND10 structures in HIV-positive cells or uninfected cells (mock) was determined and is blotted as percentage of total PML-positive cells (*n* = 100 cells/time point).

HIV infection induces a temporary localization of PML in the cytoplasm of HFFs. It has been shown in previous studies that HIV-1 infection triggers an early relocalization of PML bodies from the nucleus to the cytoplasm of infected HeLa cells [18]. To determine whether this relocalization could also explain the effect of PML on HIV reverse transcription in primary HFF cells, we analyzed the cellular localization of PML at various time points upon infection with VSV-G pseudotyped, fluorescently labeled HIV particles (HIV-MAGFP) [25]. The particles contain a Matrix-EGFP fusion protein, which allowed us to monitor the PML distribution in infected cells prior to HIV gene expression by confocal microscopy. We determined the localization of PML in uninfected cells (mock) and infected cells at various time points postinfection, starting as early as 0.5 h after inoculation with reporter virus (MOI of 3) (Figure 10). We detected dot-like GFP signals in all infected but not in mock-treated samples, suggesting the presence of cell-bound HIV-GFP virions as early as 0.5 h postinfection. At 24 h postinfection, we found Gag-EGFP reporter gene expression in almost all cells, suggesting that the GFP reporter virus used is capable of productively infecting HFF cells. In untreated cells, PML was found to be localized almost exclusively in ND10 structures within the nucleus (Figure 10A,B). In infected samples, however, we found an increasing number of cells over time showing a cytoplasmic dot-like PML staining (Figure 10B). While the percentage of these cells was relative low at 0.5 h postinfection (20%), we found the frequency of cytoplasmic PML-containing cells to be increased at 1 h and 2 h postinfection and to peak around 4 h postinfection at approximately 50%. In line with a previous publication, we found that the relocalization of PML to the cytoplasm is only temporary [18]. At 24 h postinfection only nuclear, but almost no cytoplasmic PML was detectable in HIV-GFP-positive cells. It is unclear at the moment how this relocalization works and why it takes place in only 50% of the infected cells, but, interestingly, the relocalization of PML to the cytoplasm of infected cells early during infection correlates with the early effect of PML on HIV reverse transcription. Thus, these results might be a first step in shedding light on the unknown mechanism of PML restriction described within this study.

## 4. Discussion

Within this study, we analyzed the role of PML/TRIM19 and ND10s during retroviral replication and compared the antiviral effect of PML on retroviral reporter virus infectivity in various human and murine cell types. We found that the knockdown of PML in primary human fibroblasts enhanced not only HIV-1 infectivity but also the infection of the retroviruses SIV, MLV and MPMV. Thus, our results indicate a broad antiretroviral activity of PML that is not specific to HIV-1. We knocked down PML in various cell types, including human T cell lines and myeloid cell lines, but could only detect increased HIV infectivity in human and murine fibroblasts, HFF and MEF, suggesting a cell-type specific antiretroviral effect of PML. In both cell types, we found enhanced levels of reverse transcription products in the absence of PML already 12 hours postinfection. We also found an early relocalization of PML to the cytoplasm during retroviral infection, suggesting a PML-mediated block to infection during the early phases of infection in the cytoplasm of infected cells.

Previous studies used the drug arsenic trioxide (As_2_O_3_) to analyze the effect of PML on HIV infection [18,19,20]. As As_2_O_3_ induces the degradation of PML and dissolves PML-NBs, it has been suggested that PML inhibits HIV-1 infection [18]. However, a second study did not detect any differences in HIV infection between murine WT and PML knockout cells and found that As_2_O_3_ enhanced HIV-1 infectivity independently of PML [19]. These contradictory results could be due to the known pleiotropic effects of As_2_O_3_, which has been shown to bind and modulate many cellular targets in addition to PML [32,33]. These interactions might also vary between the different cell types used in these initial studies. To determine whether arsenic treatment also leads to an enhancement of infection in HFF cells, we infected sodium arsenite (NaAsO_2_)-treated shPML and shControl HFFs with HIV reporter virus (Figure 1E). Although we found that NaAsO_2_ treatment, which has been shown to very efficiently decrease cellular PML level [30], enhanced HIV infectivity on shControl cells to the level of untreated shPML cells, we also found an enhancement of HIV infectivity on shPML HFFs upon incubation with NaAsO_2_. This finding suggests additional unspecific effects of arsenite treatment on HIV infectivity in HFFs. To avoid any further NaAsO_2_-related complications, we used shRNA-mediated PML knockdown cells or murine knockout fibroblasts to analyze the effect of PML on HIV infectivity. In contrast to Berthoux *et al.*, we saw a clear enhancement (up to 10-fold) of HIV infection in KO MEFs compared to WT MEFs. The differential outcome may be explained by the use of MEFs from different mouse strains. The MEFs used in this study, were from mice with a C57BL/6 background, whereas Berthoux *et al.* used fibroblasts from 129/SV mice [19,34]. Since the initial study did not provide expression levels of PML, it is possible that the difference in PML protein levels between WT and KO cells in their study is rather small, which might explain the absence of an antiviral effect.

In addition to murine fibroblasts, we detected enhanced retroviral infectivity in human fibroblasts expressing shRNA directed against PML. A caveat of using knockdown cells is that the knockdown of PML also changes the sub-nuclear architecture and dissolves ND10 structures. We cannot completely rule out that the down-regulation of PML abolishes the antiviral effect of another inhibitory factor localized in ND10s and therefore only indirectly blocks HIV. We therefore analyzed the influence of the knockdown of the most likely candidates for an ND10-mediated effect on retroviral infection, the permanently ND10-associated proteins Sp100 and Daxx. However, in contrast to PML, we did not detect enhanced viral infectivity in cells harboring shRNA directed against SP100 or Daxx, indicating that both proteins do not play a role in the PML-dependent inhibition of retroviral infection. To distinguish the role of PML from other ND10-associated proteins during restriction, we infected HFF cells overexpressing single PML isoforms with HIV reporter virus. Of note, testing the antiviral effect of PML by overexpression in otherwise susceptible cell lines is complicated by the fact that at least seven different isoforms of PML have hitherto been identified. In addition to the fact that the overexpression of exogenous proteins in primary cells like HFFs is rather difficult, it has been shown that foci formed by individually overexpressed PML isoforms differ from normal ND10 in terms of composition and Sp100 modification [35]. Thus, it is conceivable that multiple isoforms need to interact to efficiently inhibit retroviral infection. Nevertheless, our infection experiments showed that the overexpression of each of the single PML isoforms I to VI moderately reduces HIV reporter virus infectivity in HFFs by approximately 2- to 3-fold on average (Figure 2). However, we could not identify a specific isoform that mediates the antiretroviral effect of PML. Although we cannot completely rule out that the missing PML isoform VII accounts for the antiviral effect, the relative uniform and moderate antiviral activity of all six isoforms suggests that a common feature present in all isoforms, e.g., in the amino-terminal tripartite motif, might be important for the antiviral effect.

Recent work by Lusic *et al.* showed that PML plays a role in the transcriptional regulation of HIV during latency, presumably by interfering directly with the LTR promoter function [21]. In our study, we use single cycle reporter viruses to determine early antiviral effects during retroviral infection. The inhibitory effect of PML during HIV latency should therefore not influence our results. Nevertheless, to exclude an influence of PML on LTR promoter activity, we used a set of different lentiviral reporter constructs in our infectivity assays. In these constructs the reporter gene GFP is driven either by the HIV-1 LTR promoter, a CMV IE promoter, or the cellular EF1α promoter. We found that all three HIV reporter viruses based on the different reporter constructs are inhibited by PML to a similar extent. Although we cannot exclude that PML acts on all three of these very diverse promoters, our results suggest that the transcription of the reporter gene is not affected by PML in our single cycle infection assays. Therefore, our findings provide evidence that PML inhibits HIV replication at at least two different steps during the viral life cycle: first, at the level of transcription during HIV latency [21], and, secondly, during or prior to reverse transcription of the viral RNA genome (Figure 7 and Figure 8). Thus, our finding that already reverse transcription of incoming virus particles is hampered by PML suggests a role for PML in the cytoplasm of the infected cell during this process. And indeed, we found that PML relocalizes from the nucleus to the cytoplasm of infected cells during the early phases of retroviral replication (Figure 10). Interestingly, we also found that PML is only active against retroviruses in human or murine fibroblasts, suggesting a cell type-specific block. Although this experiment is hardly feasible at the moment due to the lack of isoform-specific antibodies, it will be very interesting in future studies to compare the expression pattern of the different PML isoforms in susceptible and non-susceptible cell types.

## 5. Conclusions

Our findings characterize the role of PML during retroviral infection in great detail and show that the PML-mediated restriction targets retroviral reverse transcription in the cytoplasm of human and murine fibroblasts. This study shows that PML contributes to the intrinsic restriction of retroviral infections in a cell type-dependent manner and therefore might provide a handle to unravel the unknown mechanism of the PML-mediated restriction of retrovirus.
